# Empagliflozin and memantine combination ameliorates cognitive impairment in scopolamine + heavy metal mixture-induced Alzheimer’s disease in rats: role of AMPK/mTOR, BDNF, BACE-1, neuroinflammation, and oxidative stress

**DOI:** 10.1007/s10787-025-01755-5

**Published:** 2025-05-05

**Authors:** Ebtsam S. Abdel-lah, Hoda S. Sherkawy, Wafaa H. Mohamed, Mariam A. Fawy, Asmaa A. Hasan, Asmaa A. Muhammed, Amira F. Taha, Abeer A. Tony, Nashwa Hamad, Marwa G. Gamea

**Affiliations:** 1 Department of Pharmacology, Faculty of Veterinary Medicine, Assiut University, Assiut, 71526 Egypt; 2 Department of Pharmacology, School of Veterinary Medicine, Badr University, Assiut, 11829 Egypt; 3 Department of Medical Biochemistry, Faculty of Medicine, Aswan University, Aswan, Egypt; 4 Department of Forensic Medicine and Toxicology, Faculty of Veterinary Medicine, Aswan University, Aswan, Egypt; 5 Department of Zoology, Faculty of Science, South Valley University, Qena, 83523 Egypt; 6 Department of Human Anatomy and Embryology, Faculty of Medicine, Aswan University, Aswan, 81528 Egypt; 7 Department of Medical Physiology, Faculty of Medicine, Aswan University, Aswan, 81528 Egypt; 8 Department of Pharmacology and Toxicology, Faculty of Pharmacy, Assiut University, Assiut, 71526 Egypt; 9 Department of Neuropsychiatry, Faculty of Medicine, Aswan University, Aswan, Egypt; 10 Department of Pathology, Faculty of Veterinary Medicine, Assiut University, Assiut, 71515 Egypt; 11 Department of Pharmacology, Faculty of Medicine, Assiut University, Assiut, 71526 Egypt; 12 Basic Medical Science Department, Badr University, Assiut, Egypt

**Keywords:** Empagliflozin, Memantine, Heavy metal mixtures, Scopolamine, Alzheimer’s disease, Pro-inflammatory cytokines, Oxidative stress

## Abstract

One of the major consequences of diabetes mellitus that has gained attention due to its rising incidence is cognitive impairment. Recent research suggested that sodium-glucose cotransporter-2 (SGLT-2) inhibitors can mitigate memory impairment linked to Alzheimer’s disease and are now being explored for their cognitive benefits. However, their mechanisms were not thoroughly studied. This research investigates the hypothesis of the neuroprotective effect of empagliflozin administration against scopolamine-heavy metal mixture (SCO + HMM)-treated Alzheimer’s rat models in comparison with memantine as a reference drug and the impact of their combination. Yet, the neuroprotective effects of memantine and empagliflozin combination against cognitive impairment have not been previously explored. This study employed adult male albino rats categorized into five groups. The impact of empagliflozin, memantine, and their concomitant administration on cognitive performance was assessed in a scopolamine and heavy metal mixture-treated Alzheimer’s disease model in rats. The assessment of rats’ cognitive behavior, memory, and spatial learning was conducted, followed by an evaluation of hippocampal brain-derived neurotrophic factor (BDNF), beta-secretase (BACE-1), oxidative stress (OS), and inflammatory marker activity. And, a western blot analysis was conducted to detect phosphorylated 5’ AMP-activated protein kinase (p-AMPK), phosphorylated mammalian target of rapamycin (p-mTOR), and heme oxygenase-1 (HO-1). Hippocampal and cerebellar histopathology were thoroughly examined, in addition to the expressions of amyloid β (Aβ). The current data demonstrate the involvement of the pAMPK/mTOR/HO-1 signaling pathway in empagliflozin neuroprotection against SCO + HMM-induced AD. In addition, it reduces AD hallmarks (Aβ and BACE1), neuro-inflammation, and oxidative stress sequelae, and enhances neurogenesis and synaptic density via BDNF. This study proposes that EMPA, especially when co-administered with other conventional anti-Alzheimer therapy, may be formulated into an innovative therapeutic strategy for the enhancement of cognitive impairments associated with neurodegenerative disorders.

## Introduction

Alzheimer’s disease (AD), commonly referred to as senile dementia, is a slowly progressing, age-related, degenerative condition of the central nervous system. Around 50 million people globally were estimated to be affected by AD in 2018, and it is anticipated that this number will extend to 152 million by 2050 (Lane et al. 2018). Thus, it is imperative to clarify the etiology and pathophysiology of AD and investigate efficient diagnostic and therapeutic approaches. Amyloid plaques, composed of β-amyloid peptide, are the main neuropathological lesions in AD brains. BACE1 is essential for the synthesis of amyloid β-peptide, which forms plaques between neurons from APP cleavage by beta- and gamma-secretases and contributes to AD. In the hippocampus, these plaques cause massive neuroinflammation, synaptic dysfunction, and cell death, all leading to early memory loss (Cole and Vassar 2007).

Brain-derived neurotrophic factor (BDNF) is considered the most recognized neurotrophin in the brain. It exhibits structural similarities with nerve growth factor and demonstrates elevated expression levels in the mammalian brain. BDNF is fundamental for the growth, differentiation, maturation, maintenance, and regeneration of several neuronal types in the CNS. Nevertheless, BDNF concentrations are diminished in AD (Zhang et al. 2015).

Many theories have been put out to explain the development of AD, even though its pathophysiology is incredibly complex. It has been observed that the brains of AD patients absorb and use significantly less glucose than those of healthy people. In addition, in AD, hypometabolism manifests before pathogenic alterations and cognitive impairment (Raut et al. 2023). In general, AD patients have altered glucose mechanisms in their brains. Interestingly, early aerobic glycolysis causes an energy imbalance in the brain due to the loss or malfunction of critical metabolic enzymes, which speeds up the pathophysiology of AD (Kleiman et al. 2022). Consequently, enhancing the brain’s glucose metabolism by targeting glycolysis dysfunctions may be helpful to prevent and treat AD (An et al. 2018).

The adenosine monophosphate (AMP)-dependent protein kinase (AMPK) has an activity that AMP controls (Salminen and Kaarniranta 2012). Nearly every mammalian tissue contains AMPK, necessary for the body’s regular energy metabolism. In organisms, activated AMPK regulates energy steadily and influences critical cell processes (Townsend and Steinberg 2023). Notably, AMPK’s primary role is to stimulate the synthesis of adenosine triphosphoric acid (ATP), which is required for most biological processes (Ke et al. 2018). AMPK has also been found to defend against cerebral metabolic stress and is essential for glucose and lipid metabolism (Hardie 2008). In the brain of a patient with Alzheimer’s disease, there is a reduction in AMPK activity, which suggests compromised mitochondrial biogenesis and function (Jornayvaz and Shulman 2010; Cai et al. 2012). AMPK plays an essential function in the deficits generated by Aβ oligomers (Assefa et al. 2020). Furthermore, it has been demonstrated that AMPK activation facilitates increased glucose absorption and improves brain energy metabolism (An et al. 2014) because it modulates transport processes and preserves cellular integrity from hypoxia (Dengler 2020). Moreover, AMPK activation may inhibit the production of the beta-secretase 1 enzyme (An et al. 2014), improve the autophagy of clumped Aβ and tau (Vingtdeux et al. 2011), decrease GSK-3beta activity, and halt oxidative stress (OS) (Assefa et al. 2020).

The mammalian target of rapamycin (mTOR) is a serine/threonine kinase composed of two complexes: mTORC1 and mTORC2. Maintaining a balance between the synthesis and degradation of proteins is crucial, depending on the requirements for differentiation and proliferation (Laplante and Sabatini 2012). mTORC1 is a sensor for various nutrients, including glucose, amino acids, energy sources (oxygen and ATP), growth factors, and certain neurotransmitters. It regulates fundamental processes such as protein synthesis, energy metabolism, lipid metabolism, autophagy, and lysosome biogenesis. mTORC2 is unresponsive to nutritional influences yet responsive to growth hormones that regulate cell viability, apoptosis, proliferation, and shape (Laplante and Sabatini 2012). The main regulators of mTOR include AMPK and phosphatidylinositol 3-kinase (PI3K)/Akt (protein kinase B) (Yang et al. 2020) and regulate the translation of proteins and cell development in response to a spectrum of environmental elements, including growth hormones, diet, and energy (Li et al. 2023).

The dysregulation of PI3K/Akt/mTOR signaling can augment the activity of glycogen synthase kinase-3beta (GSK-3beta) and lead to tau hyperphosphorylation, which produces neurofibrillary tangles (NFT) (Gupta et al. 2022). Amyloid β (Aβ), which comprises most accumulated plaques, interacts with the PI3K/Akt/mTOR pathway (Do et al. 2014). Aβ induces neurotoxicity in stem cells and neural cells by inhibiting the PI3K/Akt/mTOR pathway. Aβ oligomer primarily activates the GSK-3beta, which restricts the activity of the PI3K/Akt/mTOR pathway (Long et al. 2021). Also, the PI3K/Akt/mTOR pathway affects insulin signaling. Research indicates that insulin signaling is promoted upon insulin binding to the insulin receptor (IR). Insulin interaction results in the auto-phosphorylation of tyrosine residues inside the intracellular domain of the insulin receptor (IR), followed by the rapid phosphorylation of tyrosine residues in the C substrates 1–4 (IRS1-4) (Lavan et al. 1997). The IRS mediates insulin signaling through various pathways, with the PIK3/AKT/mTOR pathway associated with IRS1 being the most recognized. Phosphorylation of serine residues in IRS1 and IRS2 results in their dissociation from the insulin receptor (IR), leading to a reduction in tyrosine phosphorylation of IRS and the inhibition of insulin signaling attenuation (Mothe and Van Obberghen 1996).

In addition, mTOR modulates T cell differentiation by accelerating the polarization of Th17 and Th1 cells while inhibiting regulatory T cell differentiation (Delgoffe et al. 2009). Furthermore, increased mTOR activation causes an increase in inflammatory cytokines via the differentiation of type I macrophages (Li et al. 2020). Furthermore, the NLR family pyrin domain containing 3 inflammasome represents a key inflammatory pathway for mTOR activation (Saber et al. 2020) (Saber et al. 2021).

Restoring the circadian rhythms of mTOR enhancement can help with cognitive impairment and metabolic disorders. Intermittent fasting, calorie restriction, or increased physical activity can all have this effect. The aforementioned therapies are challenging to achieve in actual therapeutic practice and depend on the patient’s will. A previous study showed that SGLT-2 inhibitors can mimic the previously mentioned states by promoting catabolism, restoring mTOR cycling, and reducing cognitive impairment linked to metabolic disorders (Stanciu et al. 2021).

In this study, a combination of scopolamine and heavy metal mixture was used for the induction of cognitive impairment. Scopolamine blocks cholinergic neurotransmission, leading to memory impairment in rodents. Also, it increases the accumulation of ROS that induces OS, leading to memory impairment (Yadang et al. 2020). The detrimental effects of heavy metal intake in the neurological system include mitochondrial dysfunction resulting in ATP depletion, OS induction, and enzyme activity impairments. They also contribute to deleterious proteins formation such as β-amyloid and tau in AD via promoting the expression of APP and BACE (Islam et al. 2022).

Inhibitors of sodium-glucose cotransporter-2 (SGLT-2is) are new hypoglycemic drugs used to treat diabetes by increasing glycosuria and diuresis. SGLT-2is lower blood glucose levels; these modifications have further advantages, such as lowering body weight (Kramer and Zinman 2019). Since empagliflozin alleviated both cerebral microvascular and cognitive impairment in a mixed mouse model of diabetes mellitus and Alzheimer’s disease, there is mounting evidence that SGLT-2 inhibitors have neuroprotective potential (Hierro-Bujalance et al. 2020). It has also been claimed that empagliflozin may activate liver kinase B (LKB1) directly by phosphorylation or via sirtuin 1(SIRT1) (Lu et al. 2020). SIRT1 can activate LKB1 by augmenting its deacetylation, which causes it to be phosphorylated (Wang et al. 2018). Empagliflozin has previously been demonstrated to have an anti-inflammatory impact via SGLT-2-independent mechanisms such as intracellular AMPK activation, reduced superoxide generation, and suppression of oxidative stress (Zhou et al. 2018). In addition, it inhibited NF-κB/JNK/STAT signaling pathways, resulting in anti-inflammatory effects (Lee et al. 2021). Also, it was found to have an anti-inflammatory and cardioprotective impact due to its suppression of NF-κB in patients subjected to doxorubicin (Quagliariello et al. 2019). In addition, diabetic patients who received an injection of empagliflozin daily saw an improvement in their inflammatory profile and increase in their leukocyte antioxidant responses (Iannantuoni et al. 2019)

Notably, dapagliflozin has been shown to reverse autophagic flux reduction (Xu et al. 2021). In keeping with this, a recent study found that SGLT-2is can stimulate AMPK phosphorylation while inhibiting mTOR phosphorylation (Luo et al. 2021). Furthermore, dapagliflozin has been shown to restore defective autophagy in HK-2 cells treated with high glucose and in rats with obesity induced by a high-fat diet (Jaikumkao et al. 2021). SGLT-2 inhibitors have been shown to decrease NF-κB mediated inflammation, which in turn decreases neuronal damage and motor impairment in Huntington’s and Parkinson’s diseases, and alleviating oxidative stress, mitochondrial malfunction, and apoptosis (El-Sahar et al. 2020; Arab et al. 2021). Furthermore, SGLT-2is may inhibit acetylcholinesterase, making it a promising choice for treating AD (Wiciński et al. 2020).

According to the previously mentioned information, this study was designed to compare the neuroprotective role of EMPA as a member of SGLT-2is with the conventional standard therapy of memantine and their combination in the SCO+HMM AD rat model. Our study highlighted the impact of empagliflozin on the AMPK/mTOR autophagic machinery pathway, BDNF, and BACE1 activity, along with its anti-inflammatory and antioxidant activities.

## Material and methods

### Animals

This study employed 40 male adult Albino Wister rats, having an average weight of 200–250 g, that were acquired from the Faculty of Veterinary Medicine at Assiut University. Rats were accommodated in stainless steel enclosures within an adequately ventilated chamber, maintaining a 12-h light/dark cycle and a temperature of 25 ± 4 °C. The rats were given unrestricted access to food and water, and all efforts were made to alleviate the animals’ suffering during the experimental duration. The ethical council of the Faculty of Pharmacy at Assiut University approved the study under authorization number 05-2024-029. All experiment procedures followed the Guide for the Care and Use of Laboratory Animals.

### Drugs and chemicals

Empagliflozin powder was acquired from Jardiance, Boehringer Ingelheim International GmbH, Germany; memantine hydrochloride, 98%, was supplied from AK Scientific, USA; and scopolamine hydrobromide trihydrate, 99%, was sourced from Sigma Chemical Co., St. Louis, MO, USA. Heavy metals such as arsenic (As), cadmium (Cd), and lead (Pb) were acquired from BDH Chemicals Ltd (Poole, England).

### Induction of Alzheimer’s disease

Rats were given a combination of a heavy metal mixture and scopolamine hydrobromide trihydrate to induce cognitive impairment (Assi et al. 2023). For 28 days, scopolamine was given intraperitoneally (I.P.) at a dosage of 4 mg/kg once daily (Assi et al. 2022). During the trial, rats were administered a heavy metal mixture (HMM) in their water. The HMM comprised the following components: 3.80 ppm, Cd 0.98 ppm, and Pb 2.22 ppm (Ashok et al. 2015; Ashok and Rai 2023).

### Experimental design

Rats were assigned to five groups through random allocation (eight in each group):

Group 1: The control group was IP injected with saline for 28 days.

Group 2: The SCO/HMM group was injected IP with scopolamine (4 mg/kg/day) and received oral HMM-laced water for 28 days.

Group 3: The MEM group received the reference standard drug memantine (20 mg/kg/day, P.O) (Assi et al. 2023).

Group 4: The EMPA group was administered empagliflozin (10 mg/kg/day, P.O) (Borikar et al. 2024).

Group 5: The MEM+ EMPA group received a combination of memantine and empagliflozin (at the previously specified dosage).

Oral administration of the drugs was conducted via gavage 90 min before scopolamine administration, occurring daily for 28 days during the experimental period.

### Sample preparation

After the behavioral assessment, rats were euthanized under thiopental sodium anesthesia (50 mg/kg, IP) (Abdi-Azar and Maleki 2014). Then, their brains were carefully separated and cleaned with cold saline. The brains of each rat were split into two hemispheres. Following 48 h of fixation in 10% neutral-buffered formalin, the left hemisphere was prepared for immunohistochemistry and histopathology analysis, while the remaining brain tissues were stored at − 80 °C for p-AMPK, p-mTOR, and HO-1 western blot analysis. Each rat’s right hemisphere hippocampus was dissected, blotted dry, weighed, and stored at − 80 °C. After testing, the hippocampus samples were mixed with PBS (pH 7.4) and centrifuged for 10 min to remove any residual material before ELISA and colorimetric analysis.

### Behavioral tests

Rats were tested for behavior 45 min following their daily scopolamine treatments on days 21–28; the rats were transported to the test site 1 h before each session for adaptation. To minimize smell cues that could alter our results, all the behavioral pieces of equipment were wiped with 70% alcohol between rats.

### Morris water maze task

The MWM test in rodents evaluates hippocampal-dependent spatial memory and learning capabilities. The MWT was conducted by the methodology reported by Ali and Ahmed (2016) and Samman et al. (2023).

### Novel object recognition (NOR) test

The NORT was conducted to examine long-term and non-spatial memory. The assessment evaluates exploration behavior, recognition of objects, and memory, which are predicated on the rat’s intrinsic inclination to investigate novel objects rather than familiar ones. NORT was performed following the method outlined by Antunes and Biala (2012).

## Biochemical parameters

### Evaluation of hippocampal oxidative stress markers

The hippocampal level of malondialdehyde (MDA), an indicator of lipid peroxidation, along with reduced glutathione (GSH), was utilized to evaluate the impact of medication on the oxidant/antioxidant equilibrium in the brains of diseased rats, potentially serving as a mechanism for enhancing memory function. The hippocampus level of MDA was measured using a rat MDA ELISA kit purchased from Elabscience, Inc., Houston, Texas, USA (Cat No. E-EL-0060). Glutathione was detected using rat-reduced GSH ELISA kit obtained from CUSABIO, Houston, USA (Cat No. CSB-E12144r) using the method described by the manufacturer.

### Evaluation of pro-inflammatory cytokines

Hippocampal tumor necrosis factor-α (TNF-α) and interleukin-1β (IL-1β) concentrations were evaluated using rat TNF-α ELISA and rat IL-1β ELISA kits provided from CUSABIO, Houston, USA (Cat No. CSB-E11987r and CSB-E08055r respectively) as directed by the manufacturer (Barichello et al. 2010).

### Measurement of BDNF level in hippocampal tissue

According to manufacturer’s protocols, the BDNF concentration was assessed using an ELISA kit for rats obtained from ELK Biotechnology, USA (Cat No. ELK5459). The levels of BDNF were expressed in picograms per/mg protein (Amidfar et al. 2017).

### Determination of beta-secretase 1 (BACE-1) level

The hippocampal BACE-1 was estimated by the rat beta-secretase 1 ELISA kit obtained from My BioSource, CA, USA (Cat No. MBS2886958) according to the guidelines provided by the manufacturer (Ibrahim et al. 2022).

### Western blot assay

Western blot technique was employed to quantify the protein expression levels of P-AMPK, P-mTOR, and HO-1 by using primary antibodies, specifically anti-phospho-AMPKα (Thr172) (1:1000, Signaling Technology Cat No. 2531S) and anti-phospho-mTOR (Ser2448) (1:1000, Signaling Technology, Cat No. 2971S), along with anti-HO-1 EPR18161-128 (Cat No. ab18949) from Abcam, following the methodology described by (Hossain and Fujita 2002; Ibrahim et al. 2022).

### Histopathological investigation

Brain specimens were obtained for histological analysis, dehydrated using a graded series of ethanol, cleaned with xylene, and embedded in paraffin blocks. Subsequently, serial thin (5 µm) sections were obtained from paraffin blocks and stained by hematoxylin and eosin (H&E) stain (El-Kossi et al. 2024). The hippocampus and cerebellar cortex were analyzed using a light microscope (Olympus, CX31; Tokyo, Japan) and photographed using a digital camera (Toup view, LCMos10000KPA, China) attached to the microscope in the Department of Pathology, Faculty of Veterinary Medicine, Assiut University, Egypt**.**

### Neurohistomorphometric studies

Morphometric studies were conducted in H&E-stained brain sections. They included quantifying the number of degenerated dark pyramidal cells in CA1, CA2, and CA3 fields of cornu ammonis and the number of dark granule cells in dentate gyrus (DG) of hippocampus. Moreover, the number of dark Purkinje cells of the cerebellar cortex was also assessed. The morphometric studies were performed in 10 high-power fields/three sections/six rats from each experimental group using the ImageJ software analyzer computer system (Wayne Rasband, NIH, Bethesda, Maryland, USA) (Ismaeil et al. 2021).

### Immunohistochemical study

Immunohistochemical analysis was performed on paraffin-embedded brain sections from the hippocampus and cerebellar cortex of five animals per group to detect amyloid beta (Aβ) protein. Paraffin sections underwent deparaffinization in xylene and hydration in a descending ethanol series. Endogenous peroxidase activity was suppressed using 3% hydrogen peroxide, followed by antigen retrieval and blocking non-specific antigens. Subsequently, sections underwent incubation with anti-Aβ rabbit polyclonal primary antibody (dilution; 1:500, Bioss antibodies) at room temperature for 2 h. The primary antibody was detected utilizing a biotin-streptavidin detection system in conjunction with 3,3'-diaminobenzidine. (DAB) as the chromogen. The sections underwent counterstaining with hematoxylin, followed by ethanol dehydration, clearing with xylene, and subsequent mounting. Negative control slides were created by substituting the primary antibody with phosphate-buffered saline (PBS) (Suvarna 2012). The expression of Aβ was examined using a light microscope, revealing brown patches or plaques in the immune-stained brain sections. Subsequently, photomicrographs were captured using a digital camera.

### Statistical analysis

After the Shapiro-Wilk test for normality, one-way ANOVA and Bonferroni’s *post-hoc* multiple comparisons test were employed to evaluate the quantitative data. Mean ± standard error was used to express the results. Both Tukey’s *post-hoc* comparison testing (for behavioral tests) and Bonferroni’s *post-hoc* comparison test were employed to analyze the results of a one-way repeated measures ANOVA designed for biochemical measurements. Statistical analysis was performed utilizing GraphPad Prism, version 8.0.2. The assessment of statistical significance was conducted using a p-value less than 0.05.

## Results

### Effect of EMPA, MEM, and their combination on cognitive behavior of SCO+HMM-induced rats

#### Morris water maze (MWM) test

**Fig. 1:**
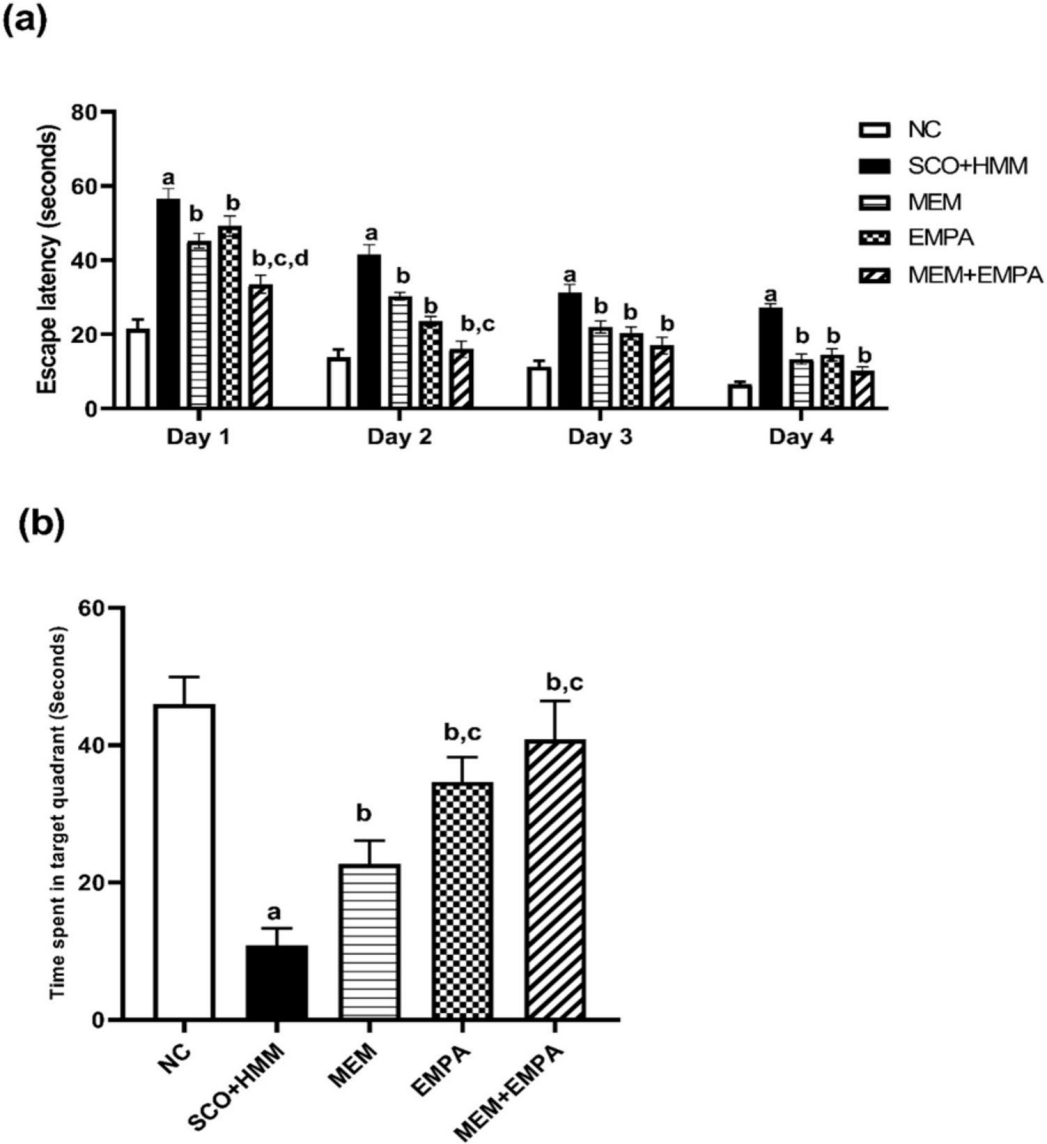
Effect of EMPA, MEM, and their combination on (**a**) escape latency (seconds) and (**b**) time spent in target quadrant (seconds) during the AD induction. Data are represented as mean ± SEM and are subjected to two-way ANOVA and then Tukey’s comparison tests **a** significant versus NC group, **b** significant versus SCO+HMM, **c** significant versus MEM, and d: significant versus EMPA. AD: Alzheimer’s disease, NC: Normal control, SCO+HMM: Scopolamine +heavy metal mixture, MEM: Memantine and EMPA: Empagliflozin (*n* = 6)

Throughout the four training days, the escape latency steadily dropped, indicating better acquisition behavior across all groups. The SCO+HMM group demonstrated a noticeably higher escape latency to reach the hidden platform with the MWM than the control group. Nonetheless, EMPA statistically significantly raised the escape latency in induced rats (*p* < 0.05). The combination of MEM and EMPA achieved more improvement in the escape latency. However, this improvement did not reach significant levels in all days (Fig. 1a) in comparison to the control group, and SCO+HMM-induced rats spent a significantly shorter duration in the target quadrant (46.00 ± 1.592 vs. 10.83± 1.014 s, respectively; *p* < 0.0001), while EMPA increased the time spent in the target quadrant in treated rats significantly not only compared to SCO+HMM group but also compared to MEM-treated rats (34.67 ± 1.476 vs. 10.83 ± 1.014 s; *p* <0.0001 for SCO +HMM group vs. 22.67± 1.382 s; *p* = 0.0001 for MEM group). When compared to the EMPA-treated group, the MEM + EMPA combination increased the amount of time spent in the target quadrant, although this increase was not statistically significant (Fig. 1b).

#### Novel object recognition (NOR) test

**Fig. 2:**
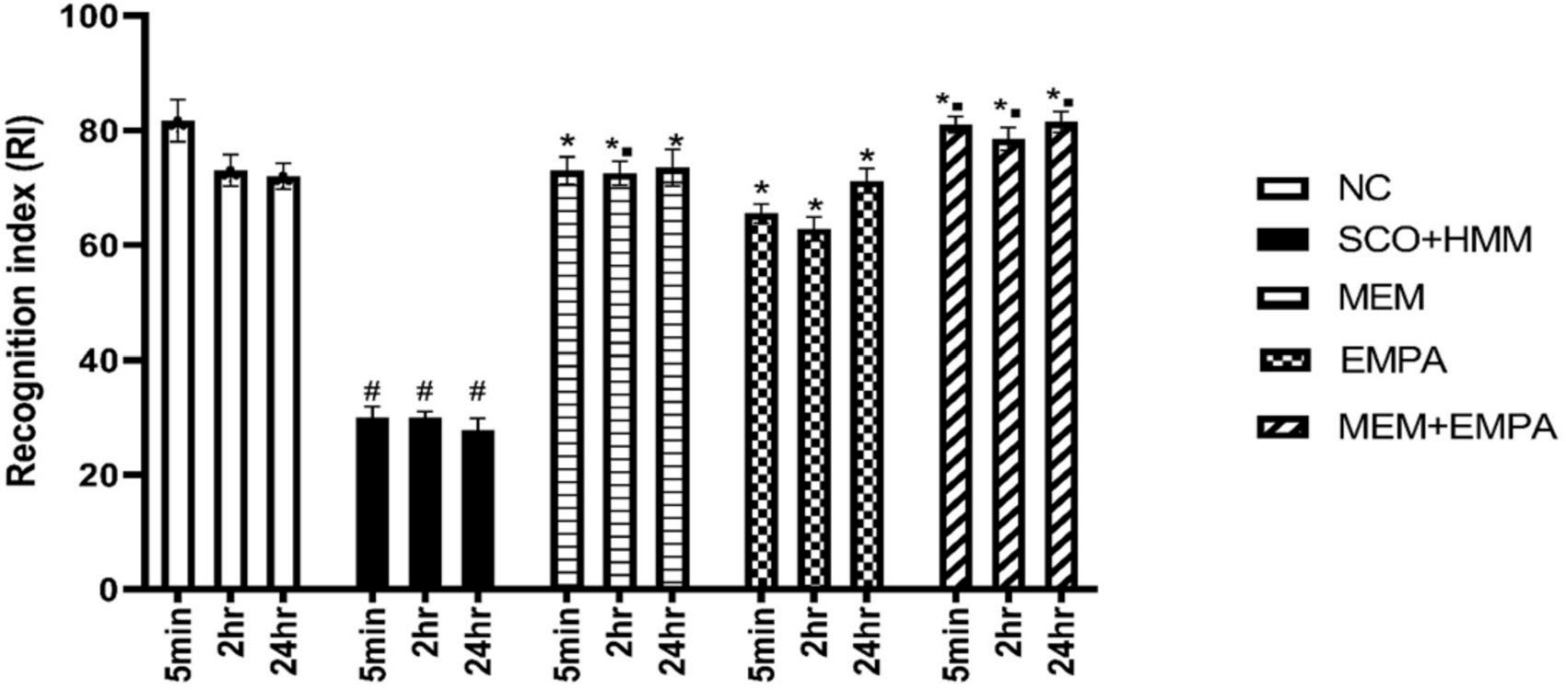
Effect of EMPA, MEM, and their combination on recognition index (RI) of novel object recognition (NOR) test in SCO+HMM-induced AD in rats. The data are expressed as mean ± SEM (*n* = 6). #: a significant difference from the NC group. *: a significant difference from the SCO+HMM group. ▪ a significant difference from EMPA group. AD: Alzheimer’s disease, NC: Normal control, SCO+HMM: Scopolamine +heavy metal mixture, MEM: Memantine and EMPA: Empagliflozin

At 5 min, 2 h, and 24 h, exploring a novel object took much longer than exploring a familiar one in the control group. The SCO+HMM group showed poor memory performance, indicating an absence of preference for a novel thing in comparison to the control group (*p* <0.0001, 5 min; 2 h; 24 h) (Fig. 2). The MEM, EMPA, and MEM+EMPA groups allocated significantly more time examining the novel object than the SCO+HMM (*p* <0.0001, 5 min; 2 h; 24 h), indicating their capacity to restore memory impairment in the AD rat model (Fig. 2). Treatment with EMPA did not demonstrate any statistically additional improvement compared to the MEM-treated group, but the MEM+EMPA combination exhibited an additive effect to the impact of the EMPA group (*p* <0.0001, 5 min; 2 h, and p < 0.05, 24 h) (Fig. 2).

## Effect of EMPA, MEM, and their combination on biochemical parameters’ levels of SCO+HMM-treated rats

### Oxidative stress markers

The administration of EMPA led to a substantial decrease (*p*<0.0001) in malondialdehyde (MDA) levels as compared to the SCO+HMM group, in which the levels of MDA were exceedingly elevated. The combination therapy by EMPA and MEM showed statistically more reduction in MDA levels than EMPA alone. Levels of glutathione (GSH) were exceedingly low in SCO+HMM-injected rats. In contrast to the SCO+HMM group, EMPA treatment exhibited a substantial increase in GSH level. Although the combination treatment provides a higher increase in GSH levels, this finding was not significant (Table 1).

**Table 1:** Impact of EMPA, MEM, and their combination treatment on brain OS biomarkers: (a) MDA and (b) GSH during the AD induction

Groups	MDA (nmol–mg)	GSH (ng–mg)
NC	0.7670 ± 0.05254	212.5 ± 9.669
SCO+HMM	9.727 ± 0.6146^a^	26.12 ± 5.417^a^
MEM	6.697 ± 0.2917^b^	75.14 ± 5.596^b^
EMPA	4.393 ± 0.3371^b,c^	136.7 ± 9.264^b,c^
MEM+EMPA	2.143 ± 0.4902^b,c,d^	163.6 ± 4.613^b,c^

Data were represented as mean ± SEM were subjected to one-way ANOVA and then Bonferroni’s *post-hoc* analysis ^a^significant versus NC group, ^b^significant versus SCO+HMM, ^c^significant versus MEM, ^d^significant versus EMPA. *AD* Alzheimer’s disease, *NC* Normal control, *SCO+HMM* Scopolamine+heavy metal mixture, *MEM* Memantine, and *EMPA* Empagliflozin, *OS* Oxidative stress (*n* = 6).

### Pro-inflammatory cytokines

**Fig. 3:**
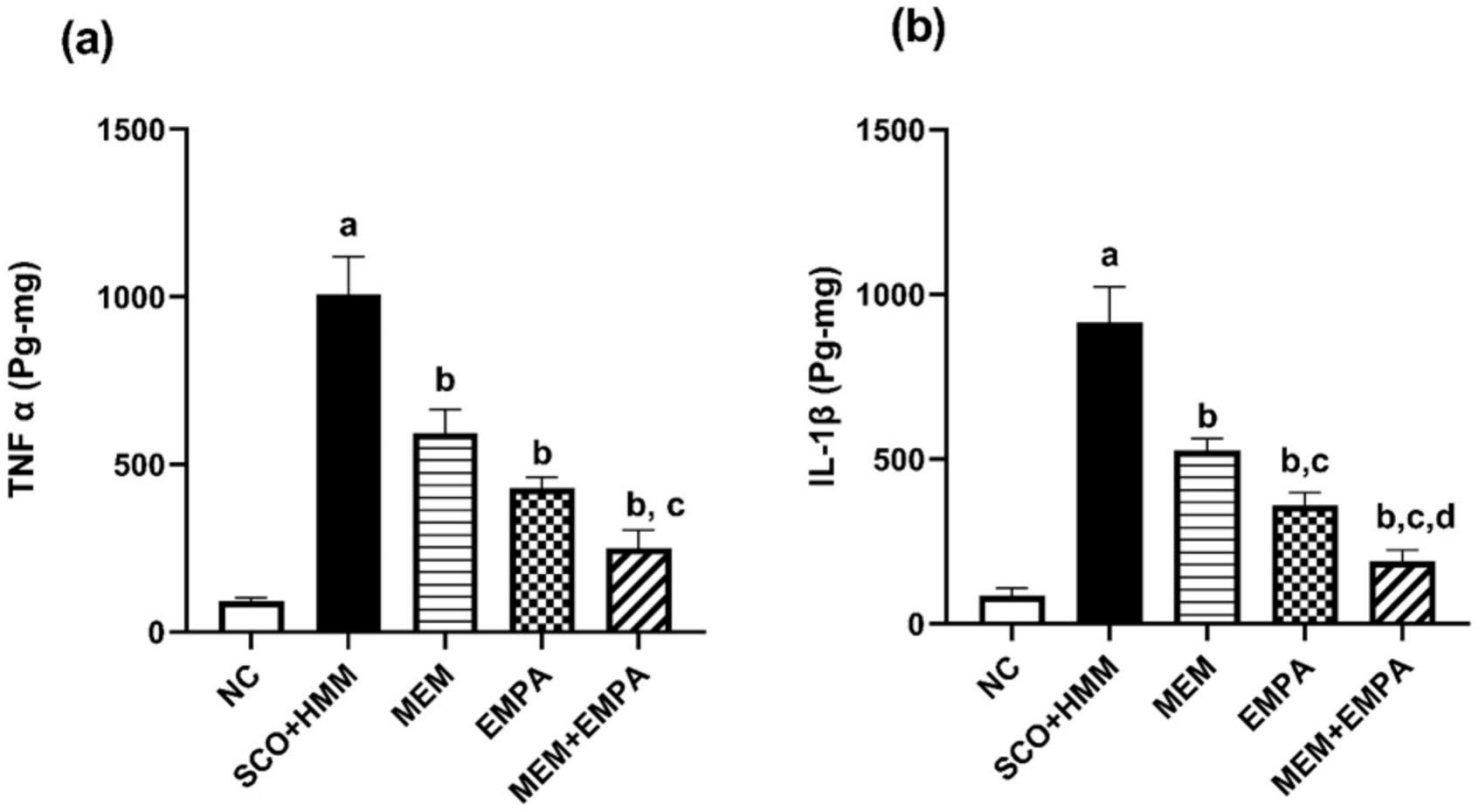
Effect of EMPA, MEM, and their combination on (**a**) TNF-α and (**b**) IL-1β during the AD induction. Data are represented as mean ± SEM and are subjected to one-way ANOVA and then Bonferroni’s *post-hoc* analysis. **a** significant versus NC group, **b** significant versus SCO+HMM, **c** significant versus MEM, and **d** significant versus EMPA. AD: Alzheimer’s disease, TNF-α: Tumor necrosis factor-alpha, IL-1β: Interleukin-1 beta, NC: Normal control, SCO+HMM: Scopolamine +heavy metal mixture, MEM: Memantine and EMPA: Empagliflozin

Induced rats revealed statistically significantly greater hippocampus TNF-α and IL-1β activity than normal control rats (1007 ± 65.25 vs. 91.95 ± 5.812 pg/mg tissue for TNF-α, respectively, *p* <0.0001; 915.4 ± 62.01 vs. 84.90 ± 13.53 pg/mg tissue for IL-1β, respectively, *p* < 0.0001). EMPA treatment statistically significantly reduced TNF-α and IL-1β activity compared to the SCO + HMM group (428.8 ± 19.25 pg/mg tissue for TNF-α, *p* <0.0001; 360.3 ± 21.31 pg/mg tissue for IL-1β, *p* <0.0001). The MEM + EMPA combination outperformed EMPA alone for IL-1β (189.6 ± 19.90 pg/mg tissue, *p* < 0.05) (Fig. 3).

### Brain-derived neurotrophic factor (BDNF)

**Fig. 4:**
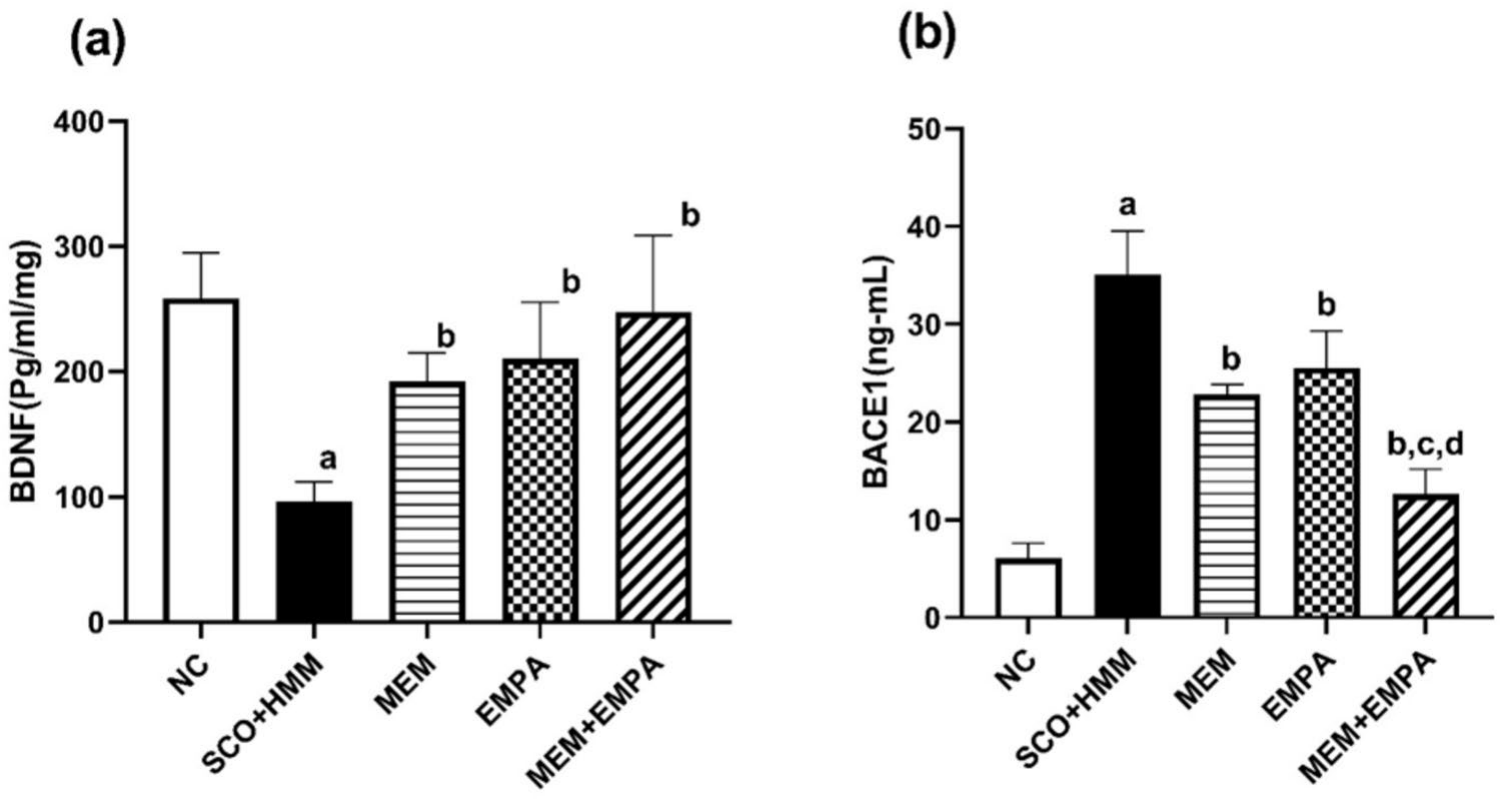
Effect of EMPA, MEM, and their combination on (**a**) BDNF and (**b**) BACE1 during the AD induction. Data are represented as mean ± SEM and are subjected to one-way ANOVA and then Bonferroni’s *post-hoc* analysis **a** significant versus NC group, **b** significant versus SCO+HMM, **c** significant versus MEM, and d: significant versus EMPA. AD: Alzheimer’s disease, BDNF: Brain-derived neurotrophic factor, BACE1: Beta-secretase 1, NC: Normal control, SCO+HMM: Scopolamine +heavy metal mixture, MEM: Memantine and EMPA: Empagliflozin

As shown in Fig. 4a, SCO+HMM produced cognitive deficits through a notable decrease of BDNF levels relative to the control group (96.50 ± 6.344 vs. 258.3 ± 14.93 pg/ml/mg, respectively, *p* <0.0001). Rats receiving EMPA had statistically significantly increased levels of BDNF compared with the diseased group (210.3 ± 18.30 pg/ml/mg, *p* <0.01). Adding MEM to EMPA treatment did not achieve a more noticeable restoration at the BDNF level.

### Beta-secretase 1 (BACE1)

One of the most significant findings regarding the neuroprotective effect of EMPA is its capacity to reduce the amount of BACE1 compared to the diseased group, which had greatly raised levels (25.51± 2.191 vs. 35.05 ±2.579 ng/ml, respectively, *p* <0.05). The MEM+EMPA combination provided the most benefit, with a more significant reduction in BACE1 levels than EMPA alone (12.60 ± 1.480 ng/ml, *p* =0.0024) (Fig. 4b).

## Phosphorylated 5’ AMP-activated protein kinase, phosphorylated mammalian target of rapamycin, and Heme oxygenase-1 signaling pathways

**Fig. 5:**
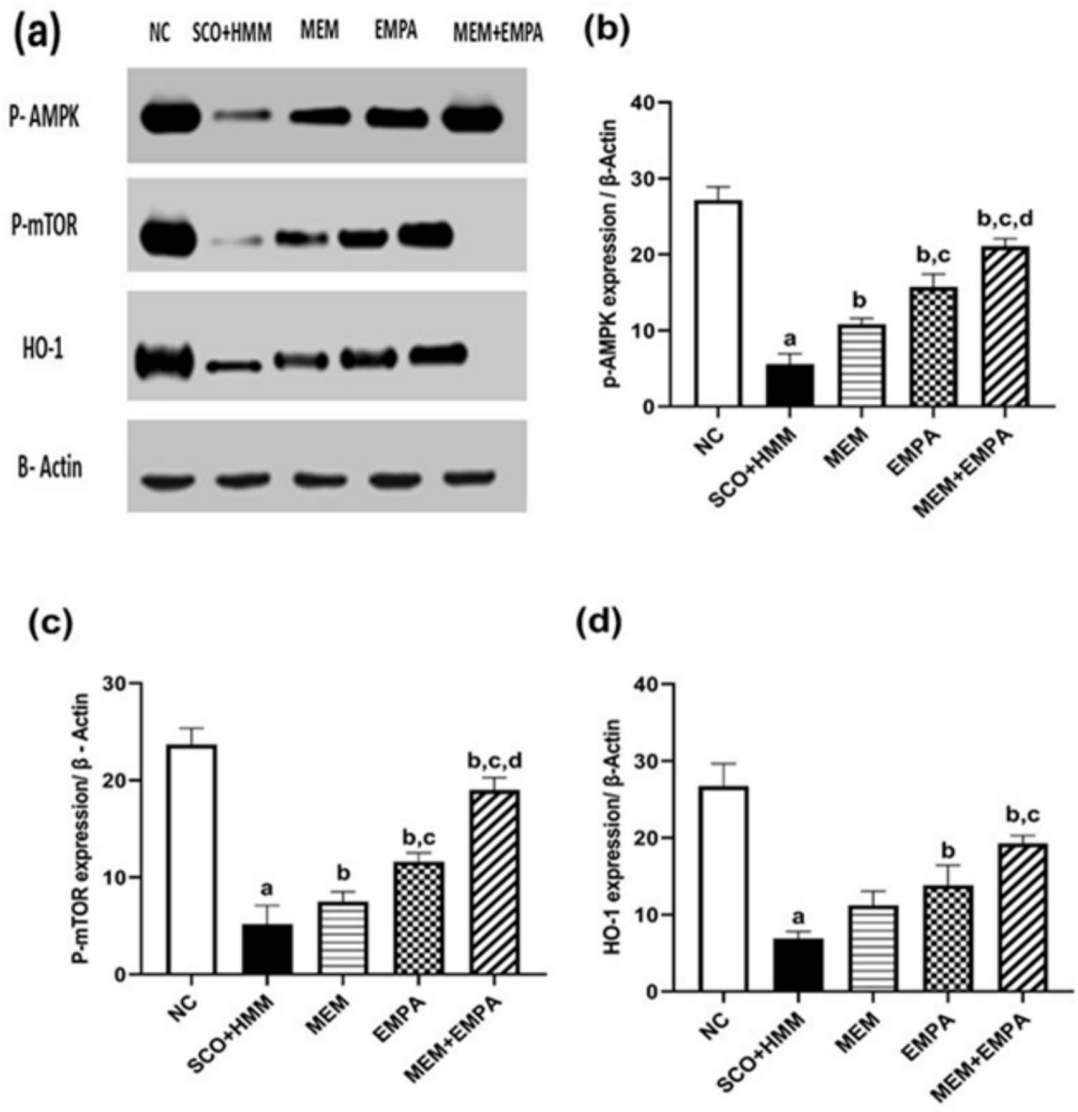
Effect of EMPA, MEM, and their combination on (**a**) western blot of all groups with data normalized to b-actin, (**b**) p-AMPK, (**c**) p-mTOR, and (**d**) HO-1 during the AD induction. Data are represented as mean ± SEM and are subjected to one-way ANOVA and then Bonferroni’s *post-hoc* analysis. **a** significant versus NC group, **b** significant versus SCO+HMM, **c** significant versus MEM, and **d** significant versus EMPA. AD: Alzheimer’s disease, p-AMPK: phosphorylated 5’ AMP-activated protein kinase, p-mTOR: phosphorylated mammalian target of rapamycin, HO-1: heme oxygenase-1, NC: Normal control, SCO+HMM: Scopolamine +heavy metal mixture, MEM: Memantine and EMPA: Empagliflozin (*n* = 3)

Western blot analysis revealed considerable (*p* < 0.0001) downregulation of p-AMPK, p-mTOR, and HO-1 in the SCO+HMM group compared to the control group. Compared to the diseased group, empagliflozin stimulated the pAMPK, pmTOR, and HO-1 signaling pathways. Compared to EMPA alone, MEM+EMPA significantly boosted p-AMPK and p-mTOR signaling pathways (Fig. 5).

## Influence of EMPA, MEM, and their combination on histopathological lesions of SCO+HMM-treated rats

**Fig. 6:**
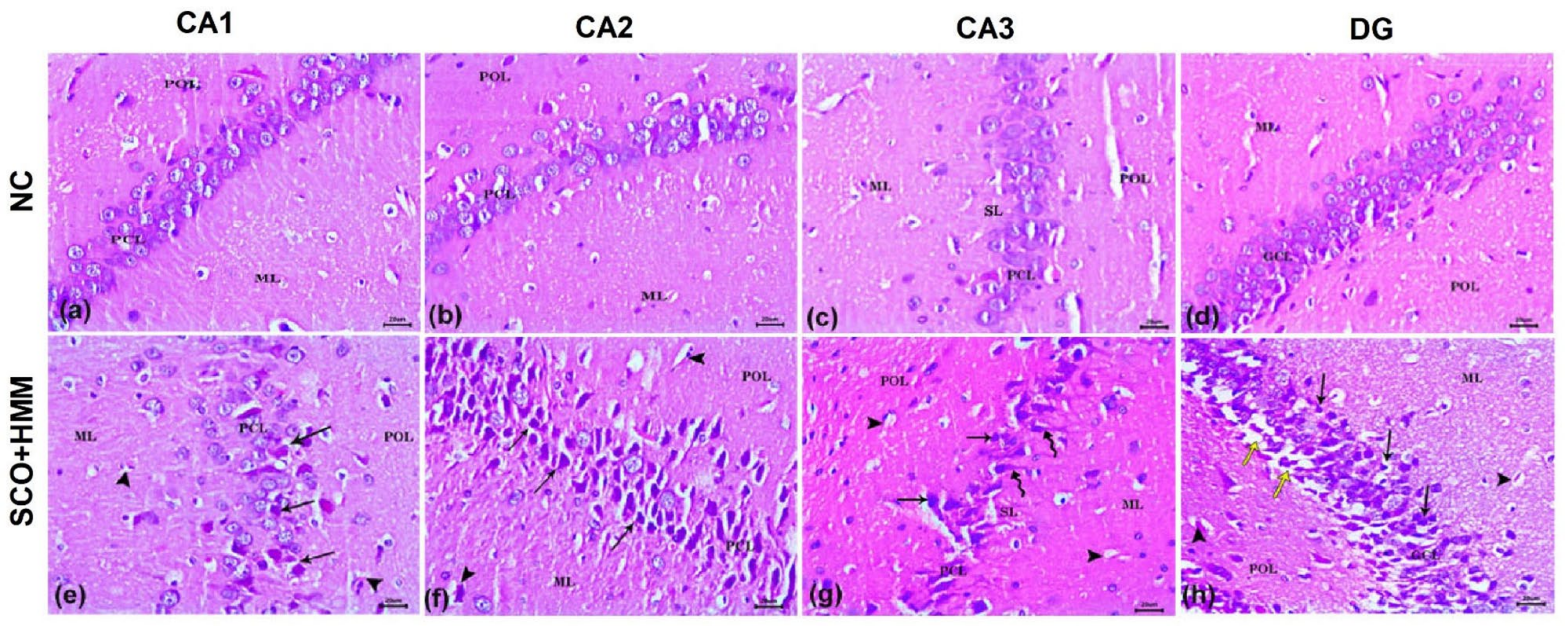
Photomicrographs of H&E-stained hippocampus sections (CA1, CA2, and CA3 fields of cornu ammonis and DG). Normal control (NC) group (**a**–**d**); polymorphic layer (POL), molecular layer (ML), and stratum lucidum (SL) as well as pyramidal cell layer (PCL) and granule cell layer (GCL) formed of perikarya with closely arranged and rounded vesicular nuclei, prominent nucleoli, and a rim of cytoplasm. Scopolamine + heavy metal mixture (SCO + HMM) group (**e**–**h**); loosely packed degenerated pyramidal and granule neurons with pyknotic nuclei, dark shrunken cytoplasm, and pericellular haloes (black arrow); dysmorphic CA3 neurons have a flame-like appearance with pointed end (wavy arrow), widening of the sub-granular zone with few immature neurons (yellow arrow), disrupted stratum lucidum (SL), and dilated blood capillaries (arrowhead). Bar= 20 μm

**Fig. 7:**
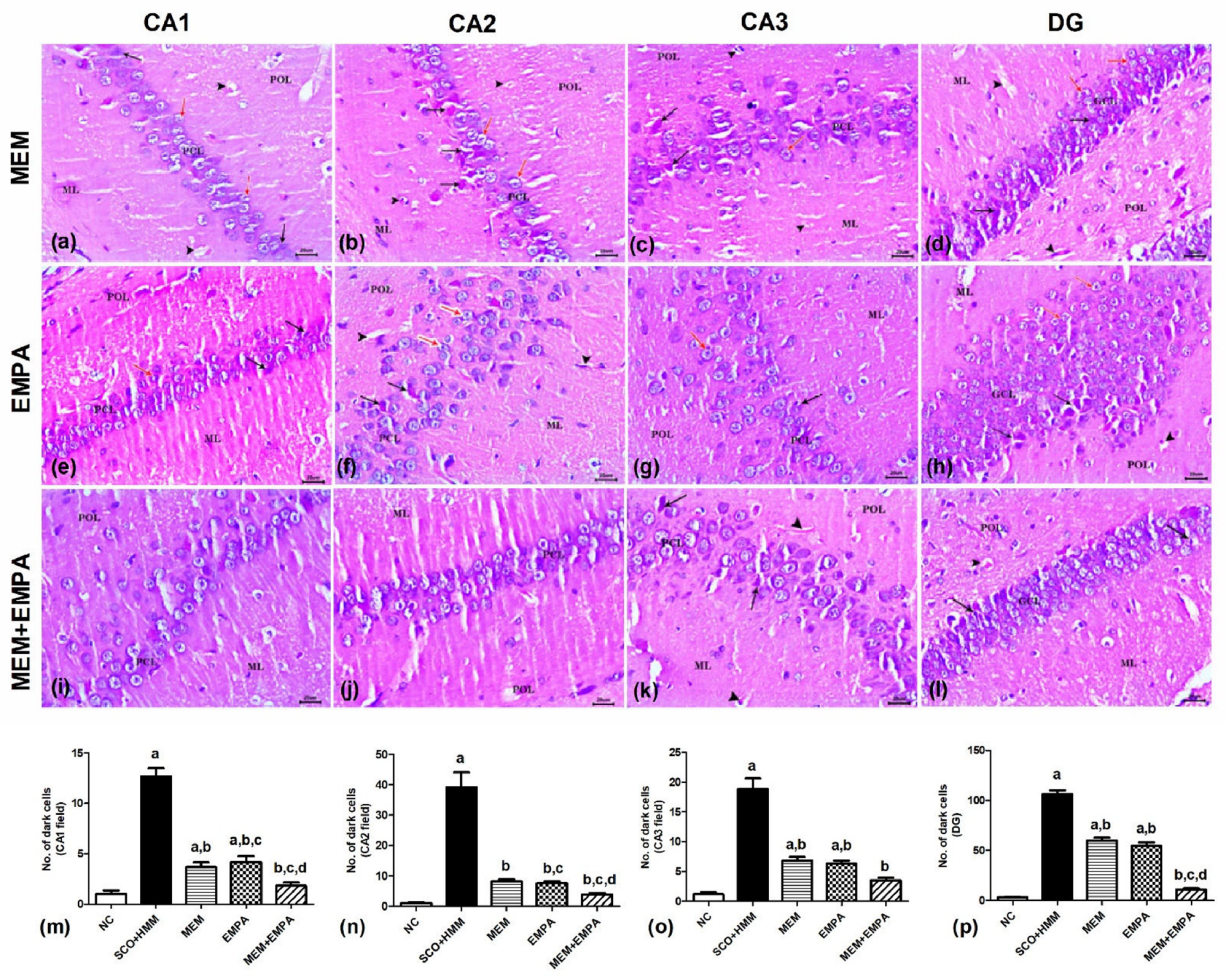
Photomicrographs of H&E-stained hippocampus sections (CA1, CA2, and CA3 fields of cornu ammonis and DG). Memantine (MEM) (**a**–**d**) and empagliflozin (EMPA) (**e**–**h**) groups; polymorphic layer (POL) and molecular layer (ML) as well as pyramidal cell layer (PCL) and granule cell layer (GCL) with somewhat regular neuronal nuclei, degenerated pyramidal and granule neurons with pyknotic nuclei, dark shrunken cytoplasm, pericellular haloes (black arrow), apparently normal neurons (red arrow), and dilated blood capillaries (arrowhead). Memantine + empagliflozin (MEM+ EMPA) group (**i**–**l**); most pyramidal (PCL) and granule (GCL) cells are well arranged with round vesicular nuclei. Note the few degenerated dark-stained cells (black arrow) and mildly dilated blood capillaries (arrowhead). Bar= 20 μm. Quantification of the number of dark pyramidal cells in CA1 (**m**), CA2 (**n**), and CA3 (**o**) fields as well as dark granule cells in DG (**p**) of all the studied groups. The values are expressed as mean ± SEM. ^a^*p* < 0.05: significant versus NC group, ^b^*p*< 0.05: significant versus SCO + HMM group, ^c^*p* < 0.05: significant versus MEM group, and ^d^*p* < 0.05: significant versus EMPA group

Examination of brain sections through histopathological analysis of the normal control rats displayed normal histomorphology of hippocampal formation. It comprises normally appeared cornu ammonis (CA) and dentate gyrus (DG). The CA was formed of CA1, CA2, and CA3 fields. Each field consisted of three layers: pyramidal cell layer (PCL), polymorphic layer (POL), and molecular layer (ML). In the PCL, the principal layer, perikarya, was closely arranged and had rounded vesicular nuclei, prominent nucleoli, and a rim of cytoplasm. In CA3, the stratum lucidum (SL) seemed normal (Fig. 6a–c). The DG was composed of three layers: granule cell layer (GCL), polymorphic layer (POL), and molecular layer (ML). The neurons of the GCL were rounded with rounded nuclei, prominent nucleoli, and thin cytoplasm. The normal appearance of the subgranular zone was noticed beneath the GCL, containing immature neurons with deeply stained oval nuclei (Fig. 6d). In contrast, the hippocampal sections of the SCO+HMM group showed marked disarrangement of PCL and GCL in CA and DG, respectively. The pyramidal cells of CA1, CA2, and CA3 were loosely packed and degenerated with pyknotic nuclei, dark shrunken cytoplasm, and pericellular haloes. CA3 revealed a dramatic reduction of cellularity and disrupted SL, and most degenerated cell bodies were dysmorphic and had a flame-like appearance with a pointed end (Fig. 6e–g). The DG showed neurodegenerative changes in granule cell bodies and widening of the subgranular zone with few immature neurons. Also, dilated blood capillaries were noticed in POL and ML (Fig. 6h). The treatment of rats with MEM moderately ameliorated the SCO+HMM-induced histopathological lesions in the hippocampus with the appearance of normal neurons accompanied by degenerated neurons and dilated blood capillaries in POL and ML (Fig. 7a–d). Similarly, EMPA-treated rats showed improved histomorphology of the hippocampus with a considerable number of apparently normal neurons, especially in CA2, CA3, and DG (Fig. 7e–h). On the other hand, the MEM+EMPA-treated group had well-arranged pyramidal and granule cells and preserved their architecture in all examined sections. A few degenerated granule cells and dilated capillaries were seen in some fields (Fig. 7i–l). Morphometric studies revealed a significant increase in the number of degenerated dark pyramidal cells in CA1, CA2, and CA3, as well as granule cells in the DG of the SCO + HMM group compared with the NC group. However, their number was significantly decreased in the treatment groups (MEM, EMPA, and MEM + EMPA) compared to the SCO + HMM group. The number of dark cells in CA1, CA2, and DG of the MEM + EMPA group was significantly decreased relative to the MEM and EMPA groups, whereas it was not significantly decreased in CA3 (Fig. 7m–p).

**Fig. 8:**
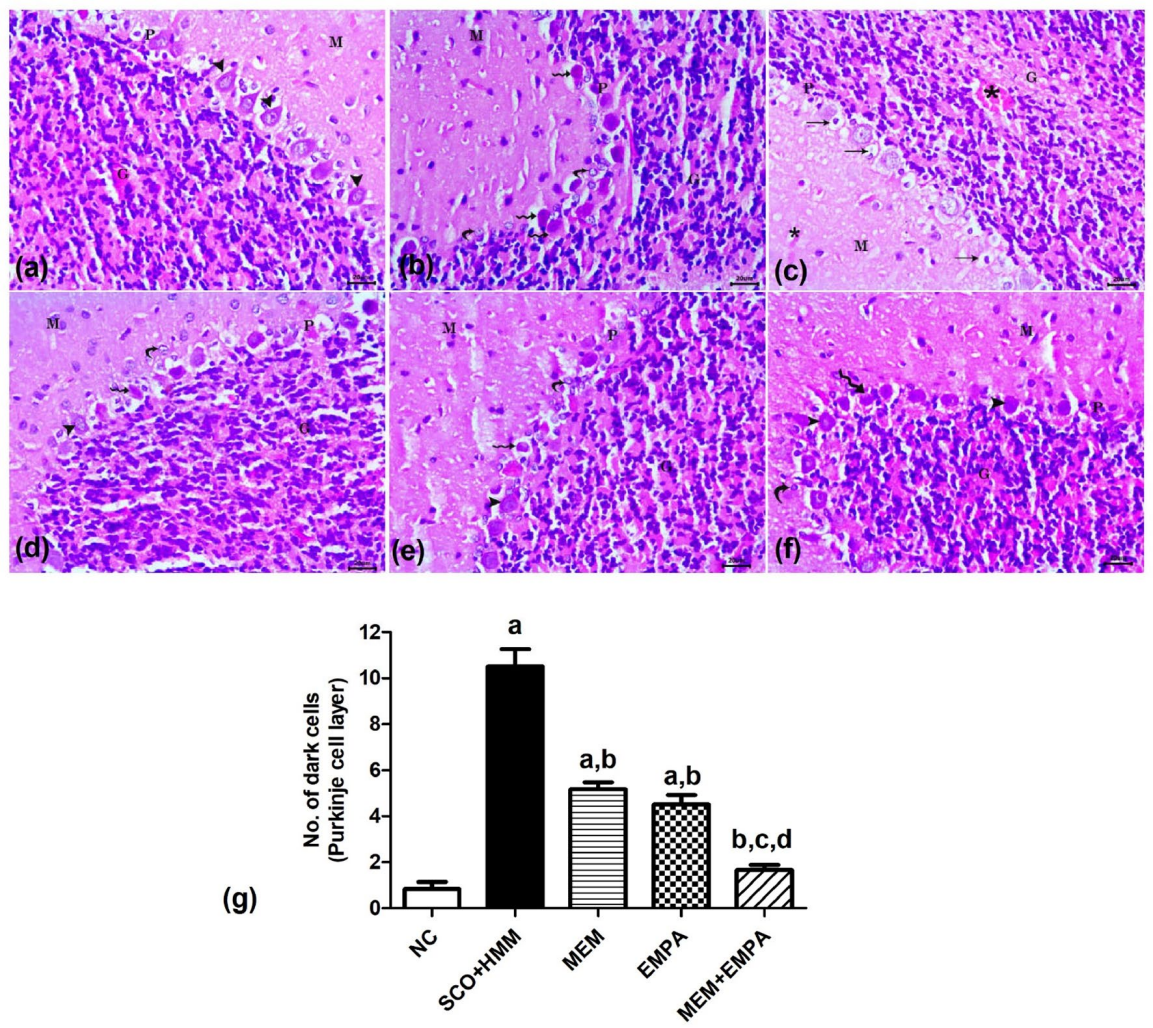
Photomicrographs of H&E-stained sections of cerebellar cortex of rats of all the studied groups. Normal control group (NC) (**a**); outer molecular layer (M), middle Purkinje cell layer (P), and an inner granule cell layer (G). Normal large flask-shaped Purkinje neurons with large vesicular nuclei (arrowhead). Scopolamine +heavy metal mixture (SCO + HMM) (**b**, **c**); the majority of Purkinje cells were disorganized and degenerated with shrunken deeply stained cytoplasm and pyknotic nuclei (wavy arrow). Purkinje cells with pyknotic nuclei surrounded by empty spaces (arrow). Marked Bergmann’s glial cell’s reaction (curved arrow). Vascular congestion (asterisk). Memantine (MEM) (**d**) and empagliflozin (EMPA) (**e**); apparently normal Purkinje neurons (arrowhead), degenerated neurons (wavy arrow), and mildly reactive glial cell (curved arrow). Memantine + empagliflozin (MEM+EMPA) (**f**); most Purkinje neurons are apparently normal (arrowhead), with few degenerated neurons (wavy arrow) and mildly reactive glial cell (curved arrow). Bar= 20 μm. Quantification of the number of dark cells in the Purkinje cell layer (**g**). The values are expressed as mean ± SEM. ^a^*p* < 0.05: significant versus NC group, ^b^*p* < 0.05: significant versus SCO+HMM group, ^c^*p* < 0.05: significant versus MEM group, and ^d^*p* < 0.05: significant versus EMPA group

H&E-stained section examination of the cerebellar cortex of the control rats revealed the normal appearance of an inner granule cell layer (G), a middle Purkinje cell layer (P), and an outer molecular layer (M). The Purkinje cell layer comprised one layer of large flask-shaped Purkinje neurons with large vesicular central nuclei (Fig. 8a). In the SCO + HMM group, the Purkinje cell layer was the most affected. There was a marked reduction of cellularity, and the majority of remaining neurons were disorganized, presented in more than one layer, and lost their characteristic flask shape with shrunken, deeply stained cytoplasm and pyknotic nuclei. Other Purkinje neurons had pyknotic nuclei surrounded by empty spaces. Moreover, a noticeable reaction of swollen Bergmann’s glial cells was detected. Molecular and granule cell layers exhibited vascular congestion (Fig. 8b, c). MEM and EMBA groups displayed moderate improvement in the histomorphology of Purkinje neurons. There were degenerated neurons and reactive Bergmann’s glial cells in addition to regular, normally seemed neurons (Fig. 8d, e). In the MEM + EMPA group, most Purkinje neurons attained their regular flask-shaped appearance with large vesicular nuclei. A few deeply stained cells, as well as mild glial cell reactions and vascular congestion in the granule cell layer, were noticed in some examined sections (Fig. 8f). Morphometrically, the SCO + HMM group displayed a significant increase in the number of degenerated dark Purkinje neurons compared to the NC group. The treated groups exhibited a significant reduction in their number relative to the SCO + HMM group. The group treated with MEM + EMPA showed a significant reduction in their number compared to other treatment groups (Fig. 8g).

## Influence of EMPA, MEM, and their combination on amyloid beta plaques of SCO+HMM-treated rats

**Fig. 9:**
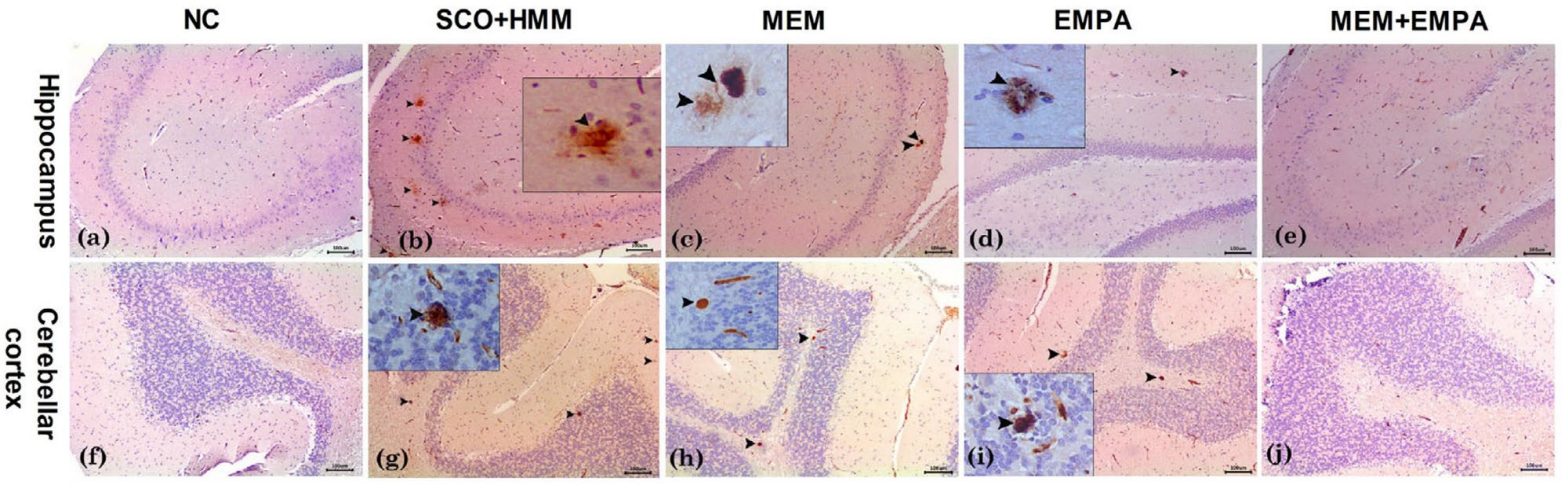
Photomicrographs of immunohistochemically stained sections of hippocampus (upper panel) and cerebellar cortex (lower panel) with anti-Aβ antibodies. **a**, **f** Normal control group (NC) showing negative immunostaining for Aβ in both hippocampus and cerebellar sections. **b**, **g** Scopolamine + heavy metal mixture group (SCO + HMM) showing many immune positive amyloid beta plaques (five in the hippocampus and four in the cerebellar cortex) of variable sizes (arrowhead). **c**, **h** Memantine group (MEM) showing two plaques in both hippocampus and cerebellar sections (arrowhead). **d**, **i** Empagliflozin (EMPA) showing one plaque in the hippocampus and two plaques in cerebellar sections (arrowhead). **e**, **j** Memantine + empagliflozin (MEM+EMPA) showing negative immunostaining in both hippocampal and cerebellar tissues. Bar= 100 μm. Insets are of high magnification

Immunohistochemical examination of amyloid beta plaques revealed a lack of positivity for anti-Aβ antibodies in the normal control group’s hippocampal and cerebellar cortical tissues (Fig. 9a, f). SCO + HMM-treated animals demonstrated the accumulation of several amyloid beta plaques, specifically five in the hippocampus and four in the cerebellar cortex, exhibiting varying sizes. The quantity of deposited plaques in the MEM group was significantly reduced to two plaques in both the hippocampus and cerebellar regions (Fig. 9c, h). In the group treated with EMPA, a significant decrease in plaque count was seen, with one plaque in the hippocampus and two plaques in the cerebellum (Fig. 9d, i). Remarkably, animals administered the MEM + EMPA combination had no plaques in all immune-stained sections of this group (Fig. 9e, j).

## Discussion

The present study investigated the neuroprotective role of EMPA in comparison with memantine as a reference drug and the effects of their combination in attenuating SCO + HMM-induced cognitive impairment in rats. EMPA was found to improve memory and learning capabilities and also affect signaling pathways such as upregulation of p-AMPK, p-mTOR, and HO-1, reducing AD hallmarks (β-amyloid and BACE1), neuro-inflammation, and oxidative stress sequelae. In addition, the results of the enhancement of neurogenesis and synaptic density via BDNF were supported by mitigating histopathological alterations. Interestingly, the rats treated with the combination exhibited a significant enhancement of cognitive impairments compared to each drug administered individually.

The scopolamine model was utilized in this study with specific modifications. Owing to its various limitations in resembling human Alzheimer’s disease, as reported by Assi et al. (2023), who enhanced the model of SCO by incorporating HMM to augment the pathogenic features of the standard SCO model. Ashok et al. (2015) discovered that rats administered with daily doses of arsenic, cadmium, and lead in drinking water exhibited significant changes resembling those of Alzheimer’s disease. The model effectively meets the criteria for Alzheimer’s disease (AD) formation, demonstrated by significant memory impairment, increased levels of brain lipid peroxidation biomarkers, elevated inflammatory cytokines, BACE1, and the presence of Aβ deposits. Conversely, GSH levels exhibited a significant decrease.

The current study demonstrated that rats in the SCO + HMM group exhibited cognitive impairment, as evidenced by their performance in the MWM and NOR tests. The results indicated that rats that received SCO + HMM exhibited an increased escape latency during the probe trial of the Morris water maze, alongside a decrease in the duration allocated to the target quadrant and the duration of exploration of the novel object, as well as the DI%. Our findings were consistent with those of Assi et al. (2023). In contrast, animals treated with EMPA exhibit a significant decrease in escape latency and allocate a significantly longer period within the specified quadrant during the MWM probe trial. In addition, the treated groups exhibited prolonged exploration of the novel object, demonstrating significantly higher DI% than the diseased rats. The results indicate that EMPA improved spatial memory and recognition memory, as well as restored cognitive function in rats induced with SCO + HMM, which is in line with our findings (Borikar et al. 2024). Also, our outcomes are consistent with the observations of other studies in animal models of dementia and T2DM (Lin et al. 2014; Sa-nguanmoo et al. 2017; Hierro-Bujalance et al. 2020; Mousa et al. 2023). Interestingly, the rats treated with the combination exhibited a significant boost in DI% relative to each drug administered individually.

Amyloid beta plaques, aging, tissue damage, metal levels, and ischemia contribute to the generation of ROS. Many investigations proved that oxidative events occur early in the illness and before pathology, indicating the function of oxidative stress and modifying drugs in AD (Wang et al. 2016). Our investigation found that SCO/HMM increased brain oxidative stress, and our research outcomes are consistent with the observations of Assi et al. (2023).

Moreover, the findings of this study indicate that EMPA significantly reduced hippocampal MDA levels while concurrently increasing GSH levels compared to SCO + HMM diseased rats. Our results align with those of Anoush et al. (2025). In addition, the study of Abdelsalam et al. (2025) indicated that EMPA displayed significant neuroprotective effects against cognitive dysfunction induced by doxorubicin, which is linked to its antioxidant properties. The combination led to a more significant decrease in the MDA level compared to EMPA alone.

In reaction to amyloid precursor protein (APP), microglial cells in the central nervous system initiate inflammatory processes by releasing cytokines, resulting in a persistent inflammation condition. This sequence contributes to the accumulation of brain plaques and expedites the progression (Dong et al. 2009; Coman and Nemeş 2017). The current investigation indicates that SCO + HMM rats exhibit impairment in memory function, coupled with markedly increased hippocampus concentrations of pro-inflammatory cytokines. In agreement with our results, Assi et al. (2023). The results indicate a relation between neuro-inflammation and cognitive impairment, highlighting its contribution to the dysregulation or increase in AChE activity, which in turn results in behavioral changes in rats exhibiting dementia. Conversely, there was a considerable drop in IL-1β and TNF-α in the hippocampus of SCO + HMM rats that received EMPA treatment. In compliance with our results, Khan et al. (2021) revealed that EMPA altered oxidative parameters (TNF-α and IL-1β) in the high fructose diet-induced hyperglycemic mice. The combination yielded a more substantial decrease in hippocampal IL-1β levels than EMPA alone.

BDNF regulates memory and synaptic plasticity. Previous investigations have found reduced serum and hippocampus BDNF in AD patients (Ng et al. 2019) . Restoration of BDNF levels has demonstrated the capacity to mitigate neuronal loss, augment cholinergic activities in the hippocampus, and enhance spatial cognition in animal models (Hu et al. 2019). Consequently, BDNF signaling pathways have been investigated as a prospective objective for the advancement of innovative therapeutic drugs for Alzheimer’s disease treatment. Our results demonstrated decreased BDNF levels in the SCO/HMM group. In line with these results, Kim et al. (2023) showed cholinergic deficits and decreased BDNF signaling in mice’s hippocampus. On the other hand, the EMPA-treated group showed improvement in BDNF levels. In accordance with our results, Borikar et al. (2024) documented the enhancement of the BDNF pathway in diabetic rats. In addition, Mousa et al. (2023) reported that EMPA improved neuroplasticity by increasing the levels of BDNF in rotenone-induced parkinsonism.

The aggregation of Aβ is the principal pathology in the advancement of neurodegeneration in AD patients. Neuronal impairment in the hippocampal areas of AD may be due to amyloid-beta buildup. BACE1 catalyzes the synthesis of β-amyloid, and the elevation of BACE1 levels in this disease offers direct and persuasive justification for developing medicines aimed at BACE1 inhibition, therefore diminishing β-amyloid and its related toxicity. Herein, SCO/HMM administration produced memory impairment and forms of Aβ plaque, increasing BACE1 levels in rat brains. In alignment with this finding, Patel et al. (2024) revealed a notable increase in Aβ deposition in the brain of the scopolamine-treated AD group. Similarly, Hernández-Rodríguez et al. (2020) demonstrated that SCO induced increased BACE1 and Aβ plaque in the hippocampus of rats. In contrast, EMPA therapy demonstrated protective effects against Aβ-mediated neuronal damage and plaque formation in SCO/HMM-induced AD rats, accompanied by a reduction in BACE1 levels. The findings align with recent studies demonstrating the efficacy of EMPA in reducing Aβ in various *in vivo* models of Alzheimer’s disease (Hierro-Bujalance and Garcia-Alloza 2024). Again, the combination gave superior effect in reducing BACE1 levels compared to each medicine provided individually.

Tau serves as the principal component of neurofibrillary inclusions of AD. When tau undergoes fibrillization, it experiences abnormal post-translational modifications that lead to reduced solubility and modified microtubule stabilizing properties. Numerous tau residues are susceptible to post-translational modifications, including phosphorylation, acetylation, ubiquitination, methylation, glycation, glycosylation, SUMOylation, oxidation, and nitration. Phosphorylation is essential for Tau’s binding to microtubules. However, the hyperphosphorylation of Tau causes its detachment from microtubules and results in aggregation (Irwin et al. 2012). Also, studies have documented the acetylation of three lysines on tau, K174, K274, and K281. Acetylation of these lysines inhibits the expression of long-term potentiation at hippocampal synapses, resulting in memory impairment, and obstructs the activity-dependent recruitment of postsynaptic AMPA-type glutamate receptors required for plasticity and mitochondrial function (Tracy and Gan 2017). However, lysine residues such as K254 are susceptible to methylation and ubiquitination. Methylation may impede Tau degradation and turnover in cells by obstructing the proteasomal degradation of Tau (Balmik and Chinnathambi 2021). Ubiquitin-modified tau clumps are additionally prevalent in the brains of individuals diagnosed with Alzheimer’s disease (AD). Researches indicate that K63-related TauO correlates with enhanced seeding activity and proliferation in human tau-expressing primary neuronal and tau biosensor cells, which is linked to compromised proteasome and lysosomal processes (Puangmalai et al. 2022).

AMPK is a crucial regulator of the signaling pathway network associated with aging and governs the aging process in organisms. Furthermore, AMPK slows cell proliferation and protects against apoptosis (Hardie 2004; Föller et al. 2009). In addition, AMPK is essential for regulating the metabolism of glucose. In AD patients’ brains, activation of AMPK, the primary rate-limiting enzyme of glycolysis, can enhance glucose uptake, control glycolysis, encourage energy metabolism, and enhance cognitive performance (Xie et al. 2023). Furthermore, the AMPK signaling system controls neuroinflammation, autophagy, oxidative stress, mitochondrial function, and other processes that contribute to the development of AD (Li et al. 2022).

Our study showed that scopolamine+HMM treatment decreased the levels of pAMPK. Consistent with our results, Kazerouni et al. (2020) discovered memory impairment and Akt and AMPK dysregulation in mice following scopolamine intraperitoneal injections. Another study revealed decreased hippocampal AMPK activation after scopolamine intraperitoneal administration in rats (Aksoz et al. 2019). This is particularly relevant to insulin signaling, as AMPK can phosphorylate insulin receptors and affect brain energy consumption (Chopra et al. 2012). Similarly, Jung et al. (2016) found reduced phosphorylation of Akt and GSK-3β in the hippocampus of scopolamine-injected animals. These changes were linked to diminished synaptic plasticity and deficits in memory. Furthermore, a study of the neuroprotective effects of AMPK was reported by Kim et al. (2013). AMPK has been demonstrated to considerably reduce the decline in acetylcholinergic neurons and alleviate scopolamine-induced memory impairment.

On the contrary, therapy with MEM increased the expression of AMPK in rat brains. Our results are in line with those of Thornton et al. (2011). Also, treatment with EMPA in our study notably enhanced the elevation of AMPK levels. The combination led to a significantly higher AMPK level than each drug alone. SGLT-2is have been shown to activate AMPK, resulting in various beneficial effects in the central nervous system, cardiovascular system, and elsewhere. Muhammad et al. (2024) reported that EMPA increased hippocampal autophagic response markedly against reserpine-induced depression in rats via the AMPK/mTOR/Beclin1/LC3B signaling pathway. Also, EMPA enhanced the AMPK/SIRT-1/PGC-1α pathway in a rat model of Parkinsonism induced by rotenone (Mohammed et al. 2024). SGLT-2 inhibitors have been shown to initiate the signaling mechanisms of antioxidant and anti-inflammatory responses via pathways that include nuclear factor-erythroid factor 2-related factor 2, AMPK, endothelial nitric oxide synthase, Akt, and HO-1. SGLT-2i therapy has been shown to significantly enhance the phosphorylation and activation of AMPK (Kamel et al. 2022). Another study reported by Amer et al. (2022) concluded that EMPA efficiently ameliorated the vigabatrin-induced cerebral toxicity via mTOR/AMPK/SIRT-1 signaling, leading to enhancement of the autophagy.

In our study, treatment with SCO+HMM led to the inhibition of p-mTOR level. Data appear inconsistent concerning the impact of various Alzheimer’s disease models on the PI3K/AKT/mTOR pathway or its individual components. Impairment of the PI3K/AKT pathway during Alzheimer’s disease progression, coupled with successive mTOR stimulation, has been shown to enhance tau phosphorylation, amyloid-beta deposition, and the cessation of autophagy, thereby exacerbating neurodegeneration (Cai et al. 2015). This demonstrates the pathogenic role of elevated mTOR levels in AD. Zhu et al. (2014) suggested a significant increase in p-mTOR levels in rats following scopolamine administration. Also, Abd Elmaaboud et al. (2023) utilized a lipopolysaccharide model, resulting in elevated p-mTOR levels, while Samman et al. (2023) used AlCl3, resulting in the suppression of p-mTOR. The contrasting outcomes regarding the effect on p-mTOR may be attributed to the different models employed to induce Alzheimer’s disease, which caused suppression of p-mTOR. In our study, the heavy metal mixture used along with scopolamine in the induction may have a role in the decrease of p-mTOR level, as several studies, which investigated the neurotoxic effect of HMM in the brain, showed a decrease in mTOR level.

Yun et al. (2019) reported that prolonged exposure to Pb in rats results in a notable drop in the expression of genes responsible for encoding IR, IRS1, PI3K, AKT2, and mTOR within the hippocampus, indicating a disruption of the insulin signaling pathway in a brain impacted by Pb. Further inquiry suggests that exposure to lead could result in oxidative harm and stimulate the generation of significant quantities of ROS, which are recognized for their role in inhibiting mTOR expression (Liou and Storz 2010).

Similarly, Huang et al. (2020) demonstrated that exposure to Pb promotes inhibition of the Akt/mTOR pathway, resulting in autophagy in astrocytes. Moreover, autophagy is vital in activating lead-induced astrocytes, subsequently enhancing inflammatory cytokines and contributing to oxidative stress. Consequently, the augmentation of autophagy in astrocytes could serve as the underlying mechanism for the neurotoxic effects associated with lead exposure. Also, Sui et al. (2015) found that the levels of p70S6K and pS6, indicators of mTORC1 activity, exhibited a gradual decline following Pb2+ treatment. The results indicate that Pb2+ has an inhibitory effect on mTORC1 activity. Also, Qi et al. (2014) showed that arsenic treatment inhibited mTOR, which activated autophagy in a systematic model of arsenic-induced cell transformation.

In our study, we observed that empagliflozin increased the levels of mTOR expression in the rats’ brains. The connection between AMPK phosphorylation and mTOR remains a subject of debate. Amino acids have been reported to activate AMPK simultaneously with mTOR (Dalle Pezze et al. 2016). On the contrary, some studies have demonstrated that EMPA showed enhanced AMPK and reduced mTOR (Amer et al. 2022); however, our study results are in line with Samman et al. (2023) and Chen et al. (2020). They discovered that dapagliflozin increased p-mTOR concentration in treated groups, indicating an increase in the levels of rapamycin-insensitive companion target and improved RICTOR and mTOR interaction. Also, dapagliflozin enhanced the phosphorylation of Akt, implying that mTORC2 is specifically activated, which promotes glucose absorption, glycolysis, and cell survival. This disagreement can be related to the heterogeneity in animal models. mTOR is renowned for its vital involvement in cell growth, metabolism, synaptic plasticity, and spatial learning; thus, any modifications in mTOR activity, whether an increase or a decrease, profoundly impact brain function.

Heme oxygenase (HO) is an enzyme that is highly conserved and implicated in various diseases. HO acts as a crucial enzyme in the breakdown of endogenous iron protoporphyrin heme, leading to the production of carbon monoxide (CO), biliverdin (BV), and ferrous ions (Fe2+), all of which perform a crucial function in sustaining hemostatic balance. Heme is essential for the survival of most organisms. In addition, in the brain, redox hemostasis affects development, aging, and neurological disorders (Franco and Vargas 2018). The by-products of HO in the brain encompass antioxidant activity, anti-apoptotic effects, vasodilation, and anti-inflammatory responses (Canesin et al. 2020; Bortolussi et al. 2021). Studies demonstrate that dysregulation of HO-1 correlates with neurodegeneration, encompassing Parkinson’s and Alzheimer’s diseases, brain inflammation, and disturbances in nervous system homeostasis.

This study showed that HO-1 levels dropped in the SCO/HMM group. Our results are in harmony with Gul et al. (2023) who documented a decrease in HO-1 levels in scopolamine-induced cognitive impairment in mice.

In contrast, treatment with MEM promoted the expression of HO-1, and our outcomes were in harmony with those of Lv et al. (2020) who reported that memantine increased the expression of the HO-1 antioxidant signaling pathway in acute myocardial infarction. Also, EMPA was found to elevate HO-1 levels. In line with these findings, Araujo et al. (2012) revealed that SGLT-2i raised Nrf2 protein concentration. As a result, Nrf2 augmentation is necessary for the transcription of the HO-1 gene. Also, Abdelsalam et al. (2025) reported that empagliflozin increases HO-1 in doxorubicin-induced chemobrain in rats. Our outcomes also showed that MEM and EMPA co-administration resulted in a synergistic enhancement in HO-1 expression compared to each drug alone.

## Conclusion

Taken together, the findings of this investigation indicate that EMPA markedly enhanced behavioral impairments during the MWM and NOR tests and also affected signaling pathways such as upregulation of AMPK/mTOR and HO-1, reducing AD hallmarks (Aβ and BACE1), oxidative stress, and neuroinflammation consequences. In addition to enhancing neurogenesis and synaptic density via BDNF, the results were supported by mitigating histopathological alterations. It was thus concluded that EMPA suggested the involvement of p AMPK/mTOR/HO-1-induced autophagy in EMPA neuroprotection against SCO + HMM-induced AD. It is possible that EMPA’s neuroprotective effects, which are linked to increased cell viability, encourage the preservation of the residual energy necessary for the cell’s survival and physiological processes in the CNS. In light of these results, we suggest that EMPA, especially when co-administered with other conventional anti-Alzheimer therapy, may be formulated into an innovative therapeutic strategy for enhancing cognitive impairments associated with neurodegenerative disorders.

## Data Availability

Data will be made available on request.

## Abbreviations

AD: Alzheimer’s disease
MEM: Memantine
EMPA: Empagliflozin
AMPK: AMP-activated protein kinase
BACE1: β-secretase enzyme
BDNF: Brain-derived neurotrophic factor
SGLT-2is: Sodium-glucose cotransporter-2 inhibitors
OS: Oxidative stress
LKB1: Liver kinase B
SIRT1: Sirtuin 1
GSK-3beta: Glycogen synthase kinase-3beta
Aβ: Amyloid β
IR: Insulin receptor
m-TOR: Mechanistic target of rapamycin
NFT: Neurofibrillary tangles
PI3K: Phosphatidylinositol 3-kinase
IL-1β: Interleukin-1 beta
As: Arsenic
Cd: Cadmium
DI: Discrimination index
HMM: Heavy metal mixture
MDA: Malondialdehyde
MWM: Morris water maze
NC: Negative control
NORT: Novel object recognition test
GSH: Reduced glutathione
Pb: Lead
ROS: Reactive oxygen species
SCO: Scopolamine
STL: Step-through latency
TNF-α: Tumor necrosis factor-α
